# Genome Update for Pseudomonas fluorescens Isolate SBW25

**DOI:** 10.1128/mra.00637-22

**Published:** 2023-01-18

**Authors:** Carsten Fortmann-Grote, Eric Hugoson, Joanna Summers, Loukas Theodosiou, Paul B. Rainey

**Affiliations:** a Department of Microbial Population Biology, Max Planck Institute for Evolutionary Biology, Plön, Germany; b Laboratoire Biophysique et Évolution, Institut Chimie Biologie Innovation, ESPCI Paris, Université Paris Sciences et Lettres, CNRS, Paris, France; Loyola University Chicago

## Abstract

We report a genome update for Pseudomonas fluorescens isolate SBW25. The updated genome assembly, which was derived from the original isolate, is based on PacBio long-read sequence data. It shows three minor differences, compared with the previously published genome sequence. Original annotations were merged with recent automated annotations to preserve information.

## ANNOUNCEMENT

Pseudomonas fluorescens is a Gram-negative bacterium that is commonly found in soil, in water, and on plant surfaces (1). Strain SBW25 (taxonomy identification number NCBItxid216595) is a model organism in evolution, population biology, microbe-plant interactions, and genetics. The Pseudomonas fluorescens SBW25 genome was originally sequenced at the Wellcome Sanger Institute (2). Sample collection and isolation are detailed in references 3 and 4. The Wellcome Sanger Institute assembled and annotated the 6,722,539-bp-long genome (2). The annotation has repeatedly been updated with automated annotation pipelines such as Prokka (5) and PGAP (6).

Our updated Pseudomonas fluorescens SBW25 genome is a *de novo* hybrid assembly of Pacific Biosciences (PacBio) Sequel single-molecule real-time (SMRT) long reads and Illumina NextSeq 500 short reads for our stock sample, MPB13884. MPB13884 was obtained by passaging the original strain PBR340, which was held at −80°C, through three consecutive single-colony-derived overnight 6-mL cultures grown at 28°C, representing approximately 100 to 150 generations.

High-density genomic DNA was extracted using the Nanobind CBB Big DNA kit (Circulomics). The library was prepared with the PacBio template preparation kit v1.0 (7). Initial shearing was performed with a MegaRuptor device, with size selection at 15 kbp. The raw data comprised 435,628 reads, with a mean length of 6,581 bp (coverage, 425×).

The raw PacBio reads were filtered using Filtlong (8) (options: –min_length 1000 –keep_percent 90). We used three different assemblers, i.e., Flye v2.5 (9) (options: –genome-size 6722539 –threads 15 –plasmids), miniasm v0.3 followed by minipolish v0.1.3 (10, 11) (both with option: –threads 15), and Raven v1.8 (12) (option: –threads 15). We produced the consensus assembly using Trycycler v0.4.1 (10, 13) (options: cluster –threads 15 followed by reconcile –threads 15 cluster_001).

Illumina short-read sequencing was performed at the Max Planck Institute for Evolutionary Biology on the NextSeq 500 platform. DNA was extracted with the Wizard genomic DNA purification kit A1120 (Promega). Library preparation with the Nextera XT kit (Illumina) followed the protocol described previously (14). The short-read data (49,451,604 paired-end reads between 35 and 151 bp in length, with 71% of reads with 150-bp length [coverage, 54×]) were preprocessed with fastp v0.20.1 (15) (default options for quality trimming and removal of Illumina adaptors). Pilon v1.23 (16) (default options) produced the hybrid assembly.

We aligned the old and new assemblies with MAFFT v1.4.0 (17) (default options). Compared to the old assembly, the new assembly is 139 bp shorter (6,722,400 bp versus 6,722,539 bp); the difference is caused by one 356-bp deletion (between position 3694247 and position 3694603) and two insertions, one of 3 bp (position 3448192 to position 3448195) and the other of 214 bp (position 895392 to position 895606).

To annotate the new assembly, we applied the following procedure. Public annotations for Pseudomonas fluorescens SBW25 were transferred from RefSeq (GenBank assembly accession number GCF_000009225.2), GenBank (accession number GCA_000009225.1), ENA (accession number GCA_000009225), and Sanger Institute (accession number AM181176) databases to the new assembly using Geneious Prime v2020.2.4 (https://www.geneious.com), applying the protocol described in reference 18 (using the method presented in exercise 3). These transferred annotations were loaded as relational database tables using gffutils v0.10.1 (19). Annotated regions with identical start, end, strand, and feature type were merged into single features, maintaining all distinct attributes. The merged table was serialized into gff3 format using Biopython v1.79 (20), converted into EMBL format using EMBLmyGFF3 v1.3 (21) (options: –topology circular –molecule_type genomic DNA –transl_table 11 –species Pseudomonas fluorescens –strain SBW25 –locus_tag PFLU –project_id PRJEA31229 –data_class WGS, –x PRO –use_attribute_value_as_locus_tag locus_tag –interleave_genes –version 1), and submitted to the ENA.

### Data availability.

Table 1 lists all relevant accession numbers. All scripts to produce the submitted assembly and annotation are available at https://gitlab.gwdg.de/mpievolbio-scicomp/sbw25_newgenome/-/tree/assembly_scripts.

**TABLE 1:** Genome assembly and annotation accession numbers for Pseudomonas fluorescens SBW25

Entity	Accession no.
Assembly and annotation	OV986001
PacBio raw reads	ERR8265873
Illumina raw reads	ERR9795577
BioSample	ERS379752
BioProject	PRJEA31229
